# The effect of allicin on the intestinal microbiota and production efficiency in selected farm animals

**DOI:** 10.2478/jvetres-2026-0026

**Published:** 2026-05-12

**Authors:** Aleksandra Jarosz, Kamil Drabik, Piotr Domaradzki, Magdalena Sapała, Monika Ziomek, Justyna Batkowska, Tomasz Grenda

**Affiliations:** 1 Department of Food and Feed Microbiology, National Veterinary Research Institute, 24-100 Puławy, Poland; 2 Institute of Biological Basis of Animal Production, Department of Commodity Science and Animal Raw Materials Processing, Faculty of Animal Sciences and Bioeconomy, University of Life Sciences in Lublin, 20-950 Lublin, Poland; 3 Department of Commodity Science and Animal Raw Materials Processing, Faculty of Animal Sciences and Bioeconomy, University of Life Sciences in Lublin, 20-950 Lublin, Poland; 4 Department of Food Hygiene of Animal Origin, Faculty of Veterinary Medicine, University of Life Sciences in Lublin, 20-950 Lublin, Poland

**Keywords:** allicin, intestinal microbiota, livestock industry, biological properties

## Abstract

Allicin is a sulphur-containing bioactive compound naturally synthesised in several *Allium* species, including white garlic (*Allium sativum* L.), bear garlic (*Allium ursinum* L.) and field garlic (*Allium vineale* L.). Current literature indicates that allicin exhibits a wide range of therapeutic activities, most notably antimicrobial, antiparasitic, antioxidant, antiviral and antifungal effects. Its biological action is primarily driven by two key mechanisms: rapid penetration into pathogenic cells and the induction of lethal intracellular alterations. The breadth of allicin’s biological properties has prompted growing interest in its potential applications in the livestock industry. Dietary supplementation with allicin has been associated with improved growth performance, enhanced immune function, better quality of animal-derived products and favourable modulation of the intestinal microbiota – an aspect of particular relevance because of the central role of gut microorganisms in animal health. The aim of this review is to summarise current knowledge on the biological properties of allicin, and to particularly consider its effects on intestinal microbial modulation and its potential to improve the production efficiency of livestock.

## Biological properties and biosynthesis of allicin

Allicin is a sulphur-containing, low-molecular-weight thiosulphinate naturally produced by several *Allium* species, most notably white garlic (*Allium sativum* L.), bear garlic (*Allium ursinum* L.) and field garlic (*Allium vineale* L.). Its reactive sulphur groups are responsible for the characteristic pungent aroma and flavour of garlic, and the compound’s chemical structure – diallyl thiosulphinate – imparts pronounced electrophilic properties because it contains a sulphinyl functional group that readily interacts with thiol-containing proteins and peptides (35, 48). This molecular reactivity is closely linked to allicin’s biological potency. Its low molecular weight enables rapid penetration through phospholipid bilayers, facilitating swift intracellular diffusion. Although allicin remains relatively stable under carefully controlled storage conditions, where it has a half-life of approximately 12 months, the decomposition time can vary markedly depending on temperature, solvent composition and exposure to light (50). Allicin’s physicochemical characteristics form the basis for its therapeutic potential and the methodological considerations necessary for laboratory handling.

From a biomedical perspective, the importance of allicin derives from the beneficial biological effects that its chemical reactivity confers in mammalian systems. It has been widely documented as a molecule with antimicrobial, antiparasitic, antioxidant, antifungal and antiviral properties. Numerous studies have indicated that allicin can enhance the activity of various antifungal agents, likely through thiol–disulphide exchange reactions that compromise fungal cell integrity and heighten susceptibility to oxidative stress (3, 31). Effects of allicin by itself have been reported against viral pathogens; *in vitro* experiments have demonstrated that allicin can inhibit influenza virus type B, herpes simplex virus type 1 and human cytomegalovirus, suggesting interference with viral envelope stability or disruption of redox-dependent intracellular replication pathways (22, 31). These actions are complemented by the biological effects of a broader family of garlic-derived organosulphur compounds, such as diallyl sulphide, diallyl disulphide, diallyl trisulphide, ajoene and S-allyl-cysteine. These molecules have variously been implicated in the modulation of lipid metabolism, antioxidant defence, inhibition of platelet aggregation, immunomodulatory effects and even anticancer activity, contributing to the extensive therapeutic value of garlic preparations (22, 31).

Despite the extensive knowledge of allicin’s biological functions, its biosynthesis within garlic remains only partially elucidated. The compound was first chemically characterised by Stoll and Seebeck in 1948, yet several aspects of its formation at the cellular level continue to be explored (5). The direct precursor of allicin is alliin (S-allyl-L-cysteine sulphoxide), a non-proteinogenic amino acid that accumulates in the cytosol of garlic cloves. Alliinase, the enzyme responsible for converting alliin to allicin, is stored separately within the vacuoles. This spatial separation prevents premature allicin formation, which would otherwise damage plant tissues because of its high reactivity. When plant cells are mechanically damaged – through crushing, cutting, or mastication – the previously segregated components come into contact, initiating the allicin-forming biochemical cascade (16). In the presence of water, alliinase catalyses the deamination of alliin, generating a highly unstable intermediate that undergoes pyridoxal phosphate-dependent dehydration. This reaction produces dehydroalanine and allylsulphenic acid, which spontaneously condenses at room temperature owing to its considerable chemical reactivity. Two molecules of allylsulphenic acid combine to form allicin *via* a thiosulphinate linkage (16, 32, 48). This rapid transformation explains the immediate release of allicin when garlic tissue is damaged, functioning as part of the plant’s chemical defence system against herbivores and microbial invaders.

The same chemical reactivity that enables allicin’s potent antimicrobial effects also results in significant instability, making the compound difficult to isolate, quantify and store. The thiosulphinate group within its structure renders it highly prone to degradation induced by heat, enzymatic activity, oxidation or various solvents. The susceptibility of allicin to various factors is shown in Table 1. These factors induce the ready decomposition of allicin into a diverse mixture of secondary organosulphur compounds, including diallyl disulphide, diallyl trisulphide, diallyl sulphide and ajoene. The formation of these derivatives with biological properties of their own complicates efforts to study allicin in its pure form. Analytical characterisation is further hindered by allicin’s volatility and short halflife under physiological conditions, as well as by the structural similarity of its numerous breakdown products. These challenges necessitate rapid extraction procedures and advanced analytical techniques capable of separating allicin from its degradation derivatives. High-performance liquid chromatography with ultraviolet detection and gas chromatography–mass spectrometry are therefore essential methods for reliably measuring allicin content and distinguishing it from closely related sulphur compounds formed during decomposition (37).

**Table 1.** Comparative stability of allicin under different solvent, temperature and pH conditions

Condition category	Specific condition	Observed stability of allicin	Notes and mechanistic considerations	Representative references
Solvent	Water (neutral pH)	Low	Rapid hydrolysis; formation of diallyl disulphide and diallyl trisulphide; strong reactivity with thiols.	(14, 23, 33, 45)
	Ethanol (70–95%)	Moderate–high	Reduced water activity slows decomposition; widely used for stabilisation in extracts.	(14)
	Oils / lipid solvents	Moderate	Lipophilic environment partially stabilises allicin but promotes conversion to oil-soluble sulphides.	(14, 55)
	4°C	High	Slower decomposition; recommended for short-term storage.	(23, 24)
Temperature	20–25°C	Moderate	Gradual degradation over hours to days; temperaturesensitive thiosulphinate bond.	(14)
	37°C	Low	Rapid decomposition; unsuitable for long-term assays.	(14, 55)
	≥50°C	Very low	Heat accelerates breakdown; explains loss of allicin upon cooking.	(14, 55)
	Acidic (pH < 4)	Moderate	Increased stability in acidic matrices; slower decomposition kinetics.	(14, 23)
pH	Neutral (pH 6–7.5)	Low	Major instability zone; fastest conversion into alk(en)yl sulphides.	(14)
	Mildly alkaline (pH 8–9)	Low	Base-catalysed decomposition accelerates thiosulphinate breakdown.	(14)
	Strongly alkaline (pH > 10)	Very low	Rapid, near-complete degradation; thiosulphinates unstable in alkaline environments.	(14)

These physicochemical characteristics of allicin – high reactivity, limited stability and rapid transformation into secondary sulphur compounds – strongly influence its biological availability and antimicrobial activity in complex biological systems. Consequently, the effects of allicin cannot be interpreted solely at the level of individual microorganisms but must be considered in the context of entire microbial communities, particularly within the gastrointestinal tract, where diverse aerobic and anaerobic populations coexist and interact.

## Antimicrobial properties and mechanisms of action of allicin

### Spectrum of antimicrobial activity and relevance to contemporary microbiology

The antimicrobial potential of allicin has been recognised for more than a century, and early on was observed by Louis Pasteur, who noted the inhibitory effects of garlic extracts prior to the chemical identification of allicin itself. The first modern, systematic demonstration of allicin’s antibacterial properties was presented in 1944 by Cavallito and Bailey (6), who reported that purified allicin inhibited both Grampositive and Gram-negative bacteria, including *Salmonella* spp., *Escherichia* spp., *Klebsiella* spp., *Bacillus* spp. and *Clostridium* spp. Some examples of antibacterial activity are presented in Table 2. Over time, subsequent studies have expanded this spectrum to include significant human pathogens such as *Helicobacter pylori*, the causative agent of gastric and duodenal ulceration, and even acid-resistant organisms such as *Mycobacterium tuberculosis*. Allicin-rich garlic extracts also reduce the production of several staphylococcal enterotoxins (A, B and C1), although they do not inhibit the synthesis of botulinum neurotoxins (4, 22).

**Table 2.** Some examples of the antimicrobial action of allicin matched to allicin source

Species or type of bacteria	Source of allicin
Gram-positive bacteria	
*Bacillus* spp.	extracted, pure allicin synthetic allicin
*Streptococcus* spp.	extracted, pure allicin synthetic allicin
methicillin-sensitive *Staphylococcus aureus*	synthetic and garlic-derived extract
methicillin-resistant *Staphylococcus aureus*	extract derived from garlic
Gram-negative bacteria	
*Salmonella* spp.	extracted, pure allicin enzymatically synthesised from alline
*Agrobacterium tumefaciens*	extract derived from garlic
*Escherichia coli*	extract derived from garlic
*Pseudomonas* spp.	extract derived from garlic

Interest is growing in allicin’s activity against multidrug-resistant and biofilm-forming bacteria, which constitute major challenges in clinical and veterinary settings. Pérez-Giraldo *et al*. (41) demonstrated that methicillin-resistant and methicillin-susceptible strains of *Staphylococcus epidermidis* showed similar susceptibility to allicin. Notably, subinhibitory concentrations of allicin significantly have reduced the ability of these bacteria to form biofilms, indicating a disruptive effect on microbial adhesion and colonisation processes (29, 41). Such findings underscore the potential role of allicin as a complementary antimicrobial agent capable of targeting pathogens that exhibit strong resilience against conventional antimicrobials. Additional studies have shown synergistic interactions between allicin and standard antibiotics, further enhancing bacterial susceptibility and undermining resistance mechanisms (29).

### Cellular penetration, thiol reactivity and disturbance of redox homeostasis

Allicin’s antimicrobial potency arises from its distinctive chemical reactivity. Its lipophilic nature enables rapid diffusion through biological membranes, granting immediate access to intracellular compartments (49, 56). Once inside the cell, allicin interacts readily with thiol-containing molecules because of the electrophilic sulphur atom within its S(O)-S structure. This leads to the formation of mixed disulphides with cysteine residues in proteins, altering their three-dimensional structure and impairing enzymatic activity (6, 33, 56). Because many essential bacterial enzymes depend on cysteine residues for catalytic function, this thiol modification can have profound metabolic consequences.

Proteomic studies by Leontiev *et al*. (25) highlighted the sensitivity of intracellular thiol systems to allicin. Their work demonstrated that allicin rapidly modifies glutathione (GSH), the principal low-molecular-weight thiol responsible for maintaining redox homeostasis. The reaction between allicin and GSH disrupts the intracellular redox potential and contributes to the accumulation of oxidative stress, which bacterial cells often cannot neutralise effectively. This oxidative imbalance may trigger apoptosis-like responses or accelerate cell death through oxidative damage.

Beyond its effects on redox dynamics, research has suggested that allicin can inhibit both DNA and RNA synthesis, interfering directly with replication and transcription (9, 46). While the precise molecular details remain under investigation, the cumulative evidence indicates that allicin targets multiple essential cellular processes simultaneously. This multi-targeted action may explain the rare emergence of bacterial resistance to allicin, even under repeated exposure.

### Disruption of membrane integrity and enzyme inhibition, and consequences for microbial viability

Along with intracellular thiol modification, allicin exerts a pronounced effect on bacterial cell membranes. Its interactions with lipid bilayers can alter membrane fluidity, disturb permeability and interfere with nutrient uptake mechanisms essential for bacterial survival. Furthermore, allicin inhibits a range of virulenceassociated enzymes, particularly thiol-dependent proteins such as thiol proteases, dehydrogenases and thioredoxin reductase (4. 33). These enzymes play significant roles in microbial pathogenesis, oxidative stress defence and metabolic regulation. Even partial inactivation can weaken the microorganism’s ability to withstand host defences or cause tissue damage, whereas higher concentrations of allicin broaden inhibition to additional enzymatic systems, ultimately leading to irreversible cellular dysfunction.

**Fig. 1.**
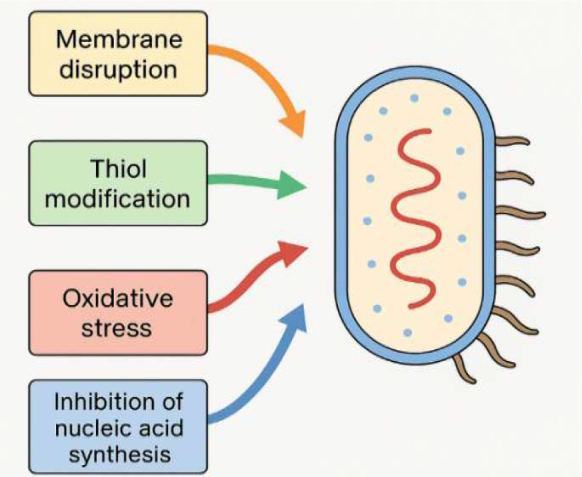
Antimicrobial mechanisms of allicin

Together, these interconnected mechanisms – membrane disruption, thiol modification, enzyme inactivation, interference with nucleic acid synthesis and induction of oxidative stress – provide a comprehensive explanation for allicin’s broad-spectrum antimicrobial effects. They also highlight why microorganisms demonstrate limited capacity to develop resistance to allicin, in contrast to their rapid adaptation to many synthetic antibiotics. As antimicrobial resistance escalates globally, allicin’s multifaceted mode of action underscores its potential utility not only in clinical medicine but also in veterinary practice and food safety interventions. The antimicrobial mechanisms of allicin action are illustrated in Fig. 1.

## The use of allicin in livestock farming

For many years, antibiotics were widely used to enhance growth rates and reduce disease incidence by suppressing bacterial infections. In response to the ban on antibiotic growth promoters, natural feed additives such as phytogenic compounds have attracted increasing attention as alternatives in livestock production. In this context, garlic-derived compounds have been widely investigated, allicin among them. While the widespread non-therapeutic administration of antibiotics for growth enhancement was initially effective in improving productivity, it had negative long-term consequences, including the selection and dissemination of antibioticresistant bacteria within the farm environment and among farmed animals. Such resistance has implications not only for animal health and production efficiency but also for food safety and public health, as resistant microorganisms can enter the food chain and potentially infect consumers. In this context, the prohibition of antibiotic growth promoters has prompted an intensive search for natural alternatives that could provide similar zootechnical benefits without contributing to resistance development. A wide range of natural feed additives, including essential oils, plant extracts, organic acids and probiotics, has therefore attracted scientific attention (7).

Among these alternatives, garlic-derived compounds, and especially allicin, stand out for their potent antimicrobial, antioxidant and immunomodulatory activities. Allicin offers several advantages: it can be produced at relatively low cost and extracted at high purity, and it demonstrates a wide range of biological effects that may benefit animal performance. Despite its potential, allicin has not yet been widely adopted as a routine supplement in livestock systems; however, a growing number of studies now document its promising applications in poultry, ruminants and, increasingly, rabbits. Evidence suggests that allicin not only enhances growth and development but may also modulate digestive processes, influence gut microbiota, improve carcass characteristics and contribute to better overall animal health. Although allicin is highly active *in vitro*, its biological effects in vivo are strongly influenced by its limited stability in the gastrointestinal tract. Allicin rapidly decomposes under physiological conditions, reacts with dietary thiols, and is transformed by intestinal and ruminal microbiota into secondary sulphur compounds with different biological activities. Consequently, the effective exposure of gut microorganisms and host tissues to intact allicin may be substantially lower than that predicted from *in vitro* assays, which likely contributes to the variability and occasional lack of measurable effects reported in animal studies (38). It should be noted that the studies of allicin’s effects on farmed animals discussed in this section differed substantially in the form of supplementation used: some experiments applied pure allicin, whereas most zootechnical studies used garlic extracts or garlic-based products containing variable and often unstable amounts of allicin. These differences have important implications for allicin’s stability, bioavailability and effective dose, and consequently for the interpretation of in vivo 2results, and are therefore explicitly distinguished throughout the following sections.

### Effects of allicin in poultry production

Most research to date has focused on poultry. Numerous studies have reported improved growth performance in broilers and laying hens following allicin supplementation, often manifested as increased feed intake and body weight gain. Although the precise mechanism remains incompletely defined, several plausible explanations have emerged. Some researchers have proposed that garlic extract stimulates appetite, thus promoting voluntary feed intake and contributing to higher weight gain (20, 44). Others have suggested more direct physiological effects: Kirubakaran *et al*. (19) proposed that bioactive sulphur compounds in garlic may stimulate the secretion of saliva and gastric juices, thereby enhancing digestion and nutrient utilisation. Contradictory findings of deleterious effects do exist, however. Certain experiments have reported minimal or no effects of allicin on poultry growth, particularly when low doses, short supplementation periods or nonstandardised garlic-derived preparations were used. These discrepancies may largely result from differences in allicin dosage, stability of the applied formulations and duration of supplementation. Several studies reporting minimal effects have used low or nonstandardised doses of garlic-derived preparations, making direct comparisons difficult and limiting the reproducibility of outcomes. It should also be noted that in several broiler studies, garlic-derived supplements have not resulted in significant improvements in growth performance or feed conversion, particularly when rapidly degradable preparations were used, suggesting limited *in vivo* persistence of allicin (20). Adjei-Mensah *et al*. (2) demonstrated that allicin supplementation in broilers reduced the incidence of diarrhoea and significantly altered haematological parameters, including globulins, MCH, RBC counts and HDL levels. These findings suggest that allicin may also modulate systemic immune and metabolic processes.

Another important line of research concerns allicin’s hypolipidaemic and hypocholesterolaemic effects. Studies involving quails, broilers and laying hens have indicated that allicin can influence lipid metabolism by inhibiting key enzymes responsible for fatty acid and cholesterol synthesis, including malic enzyme, glucose-6-phosphate dehydrogenase and fatty acid synthase (18, 54). In laying hens, dietary allicin has been associated with improved fatty acid profiles in yolks, particularly *via* increased incorporation of polyunsaturated fatty acids, and enhanced egg quality parameters, including albumen quality and egg production rates (20, 38).

### Applications in ruminant nutrition and health

In ruminants, interest in allicin centres largely on its potential to modulate the rumen microbiome. The rumen hosts a highly complex and dynamic community of microorganisms that regulate feed fermentation and nutrient assimilation and condition animal productivity. Small shifts in microbial balance can significantly influence performance, while severe dysbiosis can cause major digestive and metabolic disorders. As such, manipulating rumen microbial populations through targeted feed supplements represents a promising avenue for improving feed efficiency and production outcomes (34).

Studies of garlic-derived compounds in ruminants reveal that allicin can influence the composition and activity of rumen microbiota. Ferme *et al*. (13) cultured rumen fluid obtained from Spanish Holstein heifers in a continuous system and observed altered microbial populations following dietary garlic supplementation to the animals, notably a population decline in proteolytic *Prevotella* spp., which are associated with protein degradation. Although these findings suggest noteworthy effects on rumen fermentation pathways, the broader impacts of allicin on rumen ecology remain insufficiently explored. What is known is that rumen microbiome composition is closely tied to feed digestibility, growth performance and nitrogen metabolism (10, 11). Moreover, garlic’s bioactive constituents may contribute antiparasitic effects, supporting their potential use in managing gastrointestinal parasites in ruminants. However, *in vivo* responses in ruminants are particularly variable, and several studies have reported minimal effects of garlicbased supplementation on animal performance or rumen fermentation profiles. This variability is likely related to extensive microbial transformation and degradation of allicin in the rumen environment, which may limit its biological availability. In addition to microbial modulation, garlic-derived compounds may influence rumen fermentation efficiency and methane (CH_4_) emissions by affecting hydrogen availability and fibredegrading microbial populations. Some studies have reported reduced CH_4_ production and altered fibre fermentation at moderate supplementation levels, whereas excessive doses impaired digestibility and reduce feed efficiency. From a practical perspective, the economic effectiveness of garlic-based additives depends on dose, formulation stability and feed costs, which currently limits their large-scale application in ruminant production systems (21, 52).

### Effects of allicin and garlic extracts in rabbit production

Recent studies have indicated that rabbits may benefit substantially from allicin supplementation, both in terms of productivity and health outcomes. Omer *et al*. (39) investigated a bioactive mixture of lemon, onion and garlic juices and found marked improvements in body weight, feed conversion efficiency and carcass characteristics in treated rabbits. These enhancements were accompanied by favorable shifts in microbiological parameters, suggesting that the antimicrobial properties of garlic contribute to an improved gastrointestinal environment (39).

Immunomodulatory effects have also been demonstrated in this speces following allicin administration. El Hammed *et al*. (1) showed that dietary allicin enhanced the immune response to vaccination against *Clostridium perfringens* toxoid, leading to increased antibody titres and improved cellular immune responses. These findings underscore the capacity of allicin to support immune function in species beyond poultry, possibly through modulation of cytokine production and improved antigen presentation (1).

Additional rabbit studies have focused on physiological performance and meat quality. Pinzón Martínez *et al*. (42) reported that animals supplemented with aqueous garlic extract exhibited improved weight gain and feed efficiency, and that their meat had enhanced physical and microbiological characteristics. These results echo findings in poultry and suggest that allicin may influence lipid metabolism, oxidative processes and microbial colonisation in rabbit tissues (42). The same research group later showed that garlic extract supplementation altered caecal fermentation patterns, including reductions in total gas and carbon dioxide production, indicating shifts in microbial populations and fermentation end products (30). Because rabbits are hindgut fermenters, changes in caecal microbial ecology can have direct consequences for digestion, nutrient absorption and overall health.

Further confirmation of allicin’s antimicrobial potential comes from D’Amico et *al*. (8), who demonstrated that Phyto-L, a thiosulphonate-rich supplement derived from *Allium* spp., effectively inhibited *Escherichia coli* strains isolated from rabbits. This evidence highlights the potential of allicin-based compounds to improve microbial safety both in live animals and in meat products. Notably, discrepancies between studies using garlic extracts and those applying pure allicin suggest that the biological effects in rabbits depend strongly on formulation and stability. While extracts provide a mixture of sulphur compounds that may exert prolonged effects, pure allicin is rapidly degraded *in vivo* into sulphoxides and polysulphides. Both compositional and stability-dependent variability complicate experimental reproducibility and hamper the accumulation of a consitent evidence base. Consequently, observed outcomes are insufficiently consistent to establish formulation guidelines or justify the development of standardised commercial feeds containing allicin, limiting the practical application of the compound in rabbit production (8).

### Overall assessment and future directions

Collectively, the available evidence underscores the broad potential of allicin as a natural additive in livestock farming. Studies in poultry, ruminants and rabbits consistently point toward improvements in growth performance, digestion, lipid metabolism, immune response, product quality and gut microbial balance (Table 3). However, the variability in outcomes across studies emphasises the importance of considering multiple factors, including the method of allicin extraction, stability, purity, dosage, animal species and feeding duration.

**Table 3.** Summary of the effects of allicin supplementation in poultry, ruminants and rabbits

Species (effect category)	Observed effects	Notes and proposed mechanisms	Reference
Poultry (broilers)	↑ Growth rate; ↑ Feed intake; ↑ Body weight gain	Stimulation of appetite; enhanced digestive enzyme secretion; improved nutrient utilisation	(19, 51)
Poultry (broilers – haematology)	↓ Diarrhoea incidence; altered globulin, MCH, RBC and HDL levels	Modulation of immune response; improved metabolic homeostasis	(2)
Poultry (lipid metabolism)	↓ Cholesterol; ↓ Triglycerides; improved lipid profile	Inhibition of malic enzyme, glucose-6-phosphate dehydrogenase, and fatty acid synthase	(18, 54)
Poultry (egg production)	↑ Egg production; improved yolk polyunsaturated fatty acid profile; ↑ albumen quality	Antioxidant activity; improved nutrient assimilation; modulation of lipid metabolism	(20, 38)
Ruminants (microbiome modulation)	Changes in rumen microbial populations (↓ *Prevotella* spp.)	Antimicrobial action on proteolytic bacteria; influence on rumen fermentation	(13)
Ruminants (general productivity)	Possible improvement in feed efficiency and nutrient utilisation	Modulation of rumen microbiome linked to growth, digestibility	(10, 11, 34)
Ruminants (antiparasitic potential)	Activity against gastrointestinal parasites	Action of sulphur-containing compounds (including allicin)	(21, 52)
Rabbits (growth & carcass traits)	↑ Body weight; ↑ Feed conversion efficiency; improved carcass characteristics	Improved gut microbiology; enhanced digestion	(39)
Rabbits (immune response)	↑ Antibody titres to *C. perfringens* toxoid; ↑ lymphocyte activity	Immunomodulatory effect of allicin; stimulation of cytokine responses	(1)
Rabbits (meat quality)	Better microbial quality; improved moisture retention, tenderness	Antimicrobial properties; antioxidant effects	(42)
Rabbits (caecal fermentation)	↓ Total gas and CO2; altered fermentation metabolites	Modification of microbial communities; reduced activity of fermentative bacteria	(30)
Rabbits (antimicrobial efficacy)	Strong inhibition of *Escherichia coli* strains	Activity of thiosulphonate-rich garlic compounds	(8)

As the global livestock industry seeks sustainable alternatives to antibiotics and explores feed additives capable of enhancing production without compromising food safety, allicin emerges as a promising candidate. Further research is essential to determine optimal supplementation protocols, characterise long-term effects, and assess whether allicin may act synergistically with other phytogenic compounds or probiotic preparations (36, 44). Ultimately, a deeper understanding of allicin’s mechanisms in diverse livestock species will help determine how best to incorporate this versatile natural compound into modern, responsible and health-oriented animal-production systems.

## The effect of allicin on modification of the species composition of intestinal microbiota

The gastrointestinal tracts of animals constitute complex and dynamic microbial ecosystems in which bacteria, archaea, fungi and protozoa coexist in delicate balance. From a functional perspective, gut microbiota can be broadly divided into aerobic and facultatively anaerobic bacteria, which dominate the upper gastrointestinal tract; and strict anaerobes, which prevail in the distal intestine and play a key role in fermentation processes. Strict anaerobes, including members of *Clostridium* clusters IV and XIVa and *Bacteroides* spp., are primarily responsible for the production of shortchain fatty acids. The most abundant of these acids are acetate, propionate and butyrate, and they serve as major energy sources for enterocytes and contribute to intestinal barrier integrity and immune regulation. In healthy animals, the gut microbiota is typically dominated by beneficial and commensal bacteria, such as *Lactobacillus* and other lactic acid–producing genera, whereas potentially pathogenic genera (*e.g. Salmonella, Campylobacter, Escherichia* and *Shigella*) may be present in low abundance or transiently without causing clinical disease. The proper composition of this microbiome is essential for maintaining digestive efficiency, supporting immune functions and ensuring overall physiological stability. When this balance is disrupted, whether because of dietary shifts, environmental stressors, infectious agents or exposure to antibiotics, animals become more susceptible to gastrointestinal disorders and absorb less nutrient, and the farm notes lower animal productivity. These disturbances have implications beyond the individual animal, as many intestinal microorganisms are zoonotic, meaning that dysbiosis in livestock may adversely affect food safety and pose risks to human health.

In the search for methods to modulate the intestinal microbiota in a safe and natural way, considerable scientific attention has turned to bioactive plant-derived compounds. Allicin has emerged as a promising candidate capable of reshaping the species composition of the gut microbiome. Studies in poultry have shown that allicin administered through feed can promote favourable microbial shifts, including increased populations of beneficial *Lactobacillus* and *Streptococcus* spp., accompanied by reductions in pathogenic *Staphylococcus aureus* and *Escherichia coli* (12, 20). Some research has also reported a decrease in fungi capable of producing aflatoxins. These changes collectively contribute to improved intestinal integrity, enhanced nutrient uptake, and consequently, better weight gain and overall productivity in supplemented animals.

Although many published studies have focused on aerobic and facultatively anaerobic bacteria, researchers are increasingly examining how allicin affects strict anaerobes, especially within the *Clostridium* sensu lato group. The gut microbiome contains several important clusters of these bacteria, such as the *Clostridium coccoides* group (cluster XIVa) with approximately 21 species, the *Clostridium leptum* group (cluster IV), and various *Bacteroides* spp. (15, 26, 28). These organisms play vital roles in fermentation processes, production of short-chain fatty acids and the maintenance of mucosal health. Members of the *Clostridium* genus are Grampositive, spore-forming anaerobes that exhibit remarkable genetic and physiological diversity and inhabit soil, marine sediments and the gastrointestinal tracts of humans and animals (17). While many *Clostridium* species form part of the commensal microbiota, others are major pathogens responsible for life-threatening conditions such as botulism, gas gangrene, necrotic enteritis and other toxin-mediated diseases (40, 43, 47, 53). Their ability to produce powerful toxins and to persist in the form of hardy spores makes understanding the factors that influence their abundance in the gut critically important for animal health and public health alike.

A major limitation of currently available studies is the predominance of culture-based approaches and the limited use of high-resolution microbiome techniques, such as 16S rRNA amplicon sequencing or metagenomics. As a result, the effects of allicin on obligate anaerobes, particularly *Clostridium* clusters IV and XIVa, remain poorly characterised. This represents a critical knowledge gap, given the key metabolic and immunomodulatory roles of these bacteria in livestock species. One of the few available studies, conducted by Makuch *et al*. (27), explored the impact of allicin supplementation at 150 μg/kg and 250 μg/kg body weight on the anaerobic microbiota of quails. Notably, the higher dose of allicin significantly influenced the abundance of *Clostridium* spp. in the gastrointestinal tract. Birds receiving 250 μg/kg exhibited increased levels of *Clostridium* strains with potential probiotic value, suggesting that allicin may exert selective antimicrobial pressure that suppresses pathogenic species while promoting the growth of beneficial ones. This pattern aligns with the proposed mechanisms of allicin, which include thiol–disulphide exchange interactions that disrupt microbial metabolism in species-specific ways.

The limited number of studies investigating allicin’s effects on gut microbiota, particularly in livestock, highlights the need for more extensive research. Because microbial balance is intimately linked to production outcomes, nutrient utilisation, immune function and resistance to disease, gaining a deeper understanding of how allicin influences intestinal ecosystems is essential. Future work should focus on interactions between allicin and key microbial groups such as lactic acid bacteria, enteric pathogens, mycotoxin-producing fungi and anaerobic clostridia. Generating this knowledge will support the evidence based integration of allicin into livestock feeding strategies aimed at enhancing productivity while reducing reliance on antibiotic treatments. Although current data indicate that allicin can beneficially modify intestinal microbial communities, additional studies are required to determine optimal dosing, delivery methods, species-specific responses and the long-term implications for gut ecology and animal physiology (27).

## Conclusion

Allicin is a chemically complex and highly reactive bioactive compound synthesised by various species of garlic. As a physiologically active molecule, it displays a remarkably broad spectrum of biological activities, including antioxidant, antimicrobial and immunomodulatory effects. These properties are largely attributable to its ability to penetrate microbial cells and subsequently induce lethal damage through interactions with thiol-containing proteins and enzymes, disrupting essential metabolic pathways. Owing to this multifaceted mode of action, allicin has attracted growing interest as a natural alternative to antibiotic growth promoters, which are now prohibited in livestock farming because they contribute to antimicrobial resistance.

The scientific investigations to date have indicated that dietary supplementation with allicin can modulate several important production parameters, particularly in poultry. Reported benefits include improved growth performance, enhanced feed utilisation and better overall quality of animal-derived products such as meat and eggs. At the same time, allicin appears capable of influencing the composition and stability of the intestinal microbiome – a critical factor in digestive efficiency, immune competence and disease resistance. The gastrointestinal tracts of animals harbour intricate communities of microorganisms, dominated by facultative anaerobic genera such as *Lactobacillus, Salmonella, Campylobacter* and *Escherichia*, as well as by the obligate anaerobe genus *Clostridium*. Maintaining balanced interactions among these microbes is essential for sustaining animal health and optimal production outcomes.

Current studies suggest that allicin may support this balance by promoting beneficial bacteria such as *Lactobacillus* spp., while simultaneously suppressing harmful microorganisms including *Staphylococcus aureus, Escherichia coli* and pathogenic *Clostridium* species. Such selective modulation has important implications not only for animal performance but also for food safety, given the zoonotic potential of several intestinal pathogens. By reducing the abundance of harmful microbes, allicin could contribute to lowering the risk of enteric infections and decreasing the microbial load in animal products entering the food chain.

Despite these promising findings, no clearly established route to the practical use of allicin in livestock production exists at present. Several constraints presently militate against including allicin in commercial feed bills. Firstly, the stability of allicin is inherently low; it rapidly decomposes into secondary sulphur compounds, which complicates its isolation, purification and standardised application in feed. Secondly, the results of existing studies show considerable variability, which may be influenced by differences in garlic source material, extraction methods, dosage, animal species and production conditions. Third, while many aspects of allicin’s antimicrobial activity are known, its full mechanism of action at the cellular and ecosystem levels remains only partially understood. Importantly, the biological effects of allicin appear to be highly dose-dependent, and insufficient dosing or instability of feed additives may account for the lack of effects reported in some studies.

These gaps underscore the need for further systematic research. Future studies should focus on optimising allicin stabilisation, determining speciesspecific dosage regimens, elucidating its interactions with the gut microbiome and assessing long-term effects on animal health and productivity. Clarifying these issues will be essential for reliably integrating allicin into livestock nutrition strategies and unlocking its potential as a safe, effective and natural tool for enhancing production efficiency while reducing reliance on synthetic antimicrobials

## References

[1] Abu El Hammed W., Soufy H., El-Shemy A., Nasr S.M., Dessouky M.I. (2016). Use of allicin as feed additive to enhance vaccination capacity of Clostridium perfringens toxoid in rabbits. Vaccine.

[2] Adjei M.B., Atuahene C.C., Attoh-Kotoku V. (2015). Effects of dietary allicin on health and blood profile of broiler chickens. J Anim Sci Adv.

[3] Ankri S., Mirelman D. (1999). Antimicrobial properties of allicin from garlic. Microbes Infect.

[4] Bhatwalkar S.B., Mondal R., Krishna S.B.N., Adam J.K., Govender P., Anupam R. (2021). Antibacterial properties of organosulphur compounds of garlic *(Allium sativum)*. Front Microbiol.

[5] Borlinghaus J., Albrecht F., Gruhlke M.C.H., Nwachukwu I.D., Slusarenko A.J. (2014). Allicin: chemistry and biological properties. Molecules.

[6] Cavallito C.J., Bailey J.H. (1944). Allicin, the antibacterial principle of *Allium sativum*. I. Isolation, physical properties and antibacterial action. J Am Chem Soc.

[7] Chen J., Wang F., Yin Y., Ma X. (2021). The nutritional applications of garlic (*Allium sativum*) as natural feed additives in animals. Peer J.

[8] D’Amico F., Casalino G., Dinardo F.R., Schiavitto M., Camarda A., Romito D., Bove A., Circella E. (2023). Antimicrobial efficacy of Phyto-L, thiosulphonate from *Allium* spp. containing supplement, against *Escherichia coli* strains from rabbits. Vet Sci.

[9] Davis S.R. (2005). An overview of the antifungal properties of allicin and its breakdown products – The possibility of a safe and effective antifungal prophylactic. Mycoses.

[10] Ding H., Ao C., Zhang X. (2023). Potential use of garlic products in ruminant feeding: a review. Anim Nutr.

[11] Du H., Erdene K., Chen S., Qi S., Bao Z., Zhao Y., Wang C., Zhao G., Ao C. (2019). Correlation of the rumen fluid microbiome and the average daily gain with a dietary supplementation of *Allium mongolicum* Regel extracts in sheep. J Anim Sci.

[12] El-Ghany W.A.A. (2024). Potential effects of garlic (*Allium sativum* L.) on the performance, immunity, gut health, anti-oxidant status, blood parameters, and intestinal microbiota of poultry: an updated comprehensive review. Animals.

[13] Ferme D., Banjac M., Calsamiglia S., Busquet M., Kamel C., Avgustin G. (2004). The effects of plant extracts on microbial community structure in a rumen-simulating continuous-culture system as revealed by molecular profiling. Folia Microbiol.

[14] Fujisawa H., Suma K., Origuchi K., Kumagai H., Seki T., Ariga T. (2008). Biological and chemical stability of garlic-derived allicin. J Agric Food Chem.

[15] Guo P., Zhang K., Ma X., He P. (2020). *Clostridium* species as probiotics: potentials and challenges. J Anim Sci Biotechnol.

[16] Ilić D., Nikolić V., Nikolić L., Stankovic M., Stanojević L., Cakic M. (2011). Allicin and related compounds: biosynthesis, synthesis and pharmacological activity. Facta Univ Phys Chem Tech.

[17] Kalia V.C., Mukherjee T., Bhushan A., Joshi J., Shankar P., Huma N. (2011). Analysis of the unexplored features of rrs (16S rDNA) of the genus *Clostridium*. BMC Genomics.

[18] Khan R.U., Nikousefat Z., Tufarelli V., Naz S., Javdani M., Laudadio V. (2012). Garlic (*Allium sativum*) supplementation in poultry diets: effect on production and physiology. Worlds Poult Sci J.

[19] Kirubakaran A., Moorthy M., Chitra R., Prabakar G. (2016). Influence of combinations of fenugreek, garlic, and black pepper powder on production traits of the broilers. Vet World.

[20] Kothari D., Lee W.-D., Niu K.-M., Kim S.-K. (2019). The genus *Allium* as poultry feed additive: a review. Animals.

[21] Krstin S., Sobeh M., Braun M.S., Wink M. (2018). *Tulbaghia violacea* and *Allium ursinum* extracts exhibit anti-parasitic and antimicrobial activities. Molecules.

[22] Kyung K.H. (2012). Antimicrobial properties of allium species. Curr Opin Biotechnol.

[23] Lawson L.D., Gardner C.D. (2005). Composition, stability, and bioavailability of garlic products used in a clinical trial. J Agric Food Chem.

[24] Lawson L.D., Hunsaker S.M., Lawson L.D., Hunsaker S.M. (2018). Allicin bioavailability and bioequivalence from garlic supplements and garlic foods. Nutrients Basel.

[25] Leontiev R., Hohaus N., Jacob C., Gruhlke M.C.H., Slusarenko A.J. (2018). A comparison of the antibacterial and antifungal activities of thiosulfinate analogues of Allicin. Sci Rep.

[26] Ley R.E., Bäckhed F., Turnbaugh P., Lozupone C.A., Knight R.D., Gordon J.I. (2005). Obesity alters gut microbial ecology. Proc Nat Acad ScitU S A.

[27] Makuch A., Ziomek M., Sapała M., Drabik K., Batkowska J., Domaradzki P., Patyra E., Grenda T. (2025). The impact of allicin on the growth of *Clostridium* spp. in the digestive track of quails. Animals.

[28] Manson J.M., Rauch M., Gilmore M.S. (2008). The commensal microbiology of the gastrointestinal tract. Adv Exp Med Biol.

[29] Marchese A., Barbieri R., Sanches-Silva A., Daglia M., Nabavi S.F., Jafari N.J., Izadi M., Ajami M., Nabavi S.M. (2016). Antifungal and antibacterial activities of allicin: a review. Trends Food Sci Technol.

[30] Mariezcurrena-Berasain M.D., Mariezcurrena-Berasain M.A., Pinzón-Martínez D.L., Arzate-Serrano H.D., Ugbogu E.A., Salem A.Z.M. (2020). Influence of dietary supplementation of garlic *(Allium sativum* L.) extract on cecal productions of total gas, carbon dioxide and fermentation profiles in rabbits. Agroforest Sys.

[31] Mikaili P., Maadirad S., Moloudizargari M., Aghajanshakeri S., Sarahroodi S. (2013). Therapeutic uses and pharmacological properties of garlic, shallot, and their biologically active compounds. Iran J Basic Med Sci.

[32] Miron T., Bercovici T., Rabinkov A., Wilchek M., Mirelman D. (2004). [3H]Allicin: preparation and applications. Anal Biochem.

[33] Miron T., Rabinkov A., Mirelman D., Wilchek M., Weiner L. (2000). The mode of action of allicin: Its ready permeability through phospholipid membranes may contribute to its biological activity. Biochim Biophys Acta Biomembr.

[34] Mirzaei-Aghsaghali170 A., Syadati S.A., Fathi H., Rasouli S., Sadaghian M., Tarahomi M. (2012). Garlic in ruminants feeding. Asian J Biol Sci.

[35] Nakamoto M., Kunimura K., Suzuki J.-I., Kodera Y. (2020). Antimicrobial properties of hydrophobic compounds in garlic: allicin, vinyldithiin, ajoene and diallyl polysulphides (review). Exp Ther Med.

[36] Navidshad B., Darabighane B., Malecky M. (2018). Garlic: an alternative to antibiotics in poultry production, a review. Iran J Appl Anim Sci.

[37] Nikolic V., Stankovic M., Nikolic L., Cvetkovic D. (2004). Mechanism and kinetics of synthesis of allicin. Pharmazie.

[38] Ogbuewu I.P., Okoro V.M., Mbajiorgu E.F., Mbajiorgu C.A. (2018). Beneficial effects of garlic in livestock and poultry nutrition: a review. Agric Res.

[39] Omer H., Ahmed S., Bassuony N., Badr A., Hasanin M. (2015). Impact of adding bioactive mixture composed of lemon, onion and garlic juice on performance, carcass chracteristics and some microbiological parameters of rabbits. Adv Env Biol.

[40] Peck M.W., Smith T.J., Anniballi F., Austin J.W., Bano L., Bradshaw M., Cuervo P., Cheng L.W., Derman Y., Dorner B.G., Fisher A., Hill K.K., Kalb S.R., Korkeala H., Lindström M., Lista F., Lúquez C., Mazuet C., Pirazzini M., Stringer S.C. (2017). Historical perspectives and guidelines for botulinum neurotoxin subtype nomenclature. Toxins.

[41] Pérez-Giraldo C., Cruz-Villalón G., Sánchez-Silos R., Martínez-Rubio R., Blanco M.T., Gómez-García A.C. (2003). *In vitro* activity of allicin against *Staphylococcus epidermidis* and influence of subinhibitory concentrations on biofilm formation. J Appl Microbiol.

[42] Pinzón Martínez D.L., Mariezcurrena-Berasain M.D., Arzate Serrano H.D., Mariezcurrena-Berasain M.A., Mohamed Salem A.Z., Medina García A. (2020). Effect of the addition of aqueous extract of garlic (*Allium sativum*) to the diet of rabbits *(Oryctolagus cuniculus)* on the productivity and on the physical and microbiological quality of the meat. Rev Mexicana Cienc Pecu.

[43] Poulain B., Popoff M.R. (2019). Why are botulinum neurotoxinproducing bacteria so diverse and botulinum neurotoxins so toxic?. Toxins.

[44] Puvača N., Ljubojević D., Kostadinović L., Lukač D., Lević J., Popović S., Đuragić O. (2015). Spices and herbs in broilers nutrition: effects of garlic (*Allium sativum* L.) on broiler chicken production. Worlds Poult Sci J.

[45] Rabinkov A., Miron T., Konstantinovski L., Wilchek M., Mirelman D., Weiner L. (1998). The mode of action of allicin: trapping of radicals and interaction with thiol containing proteins. Biochim Biophys Acta Gen Subj.

[46] Reiter J., Hübbers A.M., Albrecht F., Leichert L.I.O., Slusarenko A.J. (2020). Allicin, a natural antimicrobial defence substance from garlic, inhibits DNA gyrase activity in bacteria. Int J Med Microbiol.

[47] Rood J.I., Adams V., Lacey J., Lyras D., McClane B.A., Melville S.B., Moore R.J., Popoff M.R., Sarker M.R., Songer J.G., Uzal F.A., Van Immerseel F. (2018). Expansion of the *Clostridium perfringens* toxin-based typing scheme. Anaerobe.

[48] Salehi B., Zucca P., Orhan I.E., Azzini E., Adetunji C.O., Mohammed S.A., Banerjee S.K., Sharopov F., Rigano D., Sharifi-Rad J., Armstrong L., Martorell M., Sureda A., Martins N., Selamoğlu Z., Ahmad Z. (2019). Allicin and health: a comprehensive review. Trends Food Sci Technol.

[49] Sarvizadeh M., Hasanpour O., Naderi Ghale-Noie Z., Mollazadeh S., Rezaei M., Pourghadamyari H., Masoud Khooy M., Aschner M., Khan H., Rezaei N., Shojaie L., Mirzaei H. (2021). Allicin and digestive system cancers: from chemical structure to its therapeutic opportunities. Front Oncol.

[50] Savairam V.D., Patil N.A., Borate S.R., Ghaisas M.M., Shete R.V. (2023). Allicin: a review of its important pharmacological activities. Pharmacol Res Mod Chin Med.

[51] Sheoran N., Kumar R., Kumar A., Batra K., Sihag S., Maan S., Maan N.S. (2017). Nutrigenomic evaluation of garlic (*Allium sativum*) and holy basil (*Ocimum sanctum*) leaf powder supplementation on growth performance and immune characteristics in broilers. Vet World.

[52] Strickland V.J., Krebs G.L., Potts W. (2009). Pumpkin kernel and garlic as alternative treatments for the control of *Haemonchus contortus* in sheep. Anim Prod Sci.

[53] Uzal F.A., Uzal F.A., Songer J.G., Prescott J.A., Popoff M.R. (2016). Clostridial Diseases of Animals.

[54] Wang G.-C., Han L.-L., Wang J., Lang W.-N., Pan C.-Y., Li Y.-F. (2014). Effects of allicin on lipid metabolism and antioxidant activity in chickens. J Northeast Agric Univ.

[55] Yu T.-H., Wu C.-M. (1989). Stability of allicin in garlic juice. J Food Sci.

[56] Zhou Y., Li X., Luo W., Zhu J., Zhao J., Wang M., Sang L., Chang B., Wang B. (2022). Allicin in digestive system cancer: from biological effects to clinical treatment. Front Pharmacol.

